# Reflections of Nurse Leaders on Optimising Organisational Culture in Aged Care in Rural and Regional Australia: A Critical Discourse Analysis

**DOI:** 10.1155/jonm/1474638

**Published:** 2026-05-30

**Authors:** Deborah Magee, Marguerite Bramble, Samantha Jakimowicz, Holly Randell-Moon, Karen Francis

**Affiliations:** ^1^ School of Nursing, Midwifery and Healthcare Sciences, Charles Sturt University, Panorama Avenue, Bathurst, 2795, New South Wales, Australia, csu.edu.au; ^2^ School of Indigenous Australian Studies, Charles Sturt University, 8 Tony McGrane Place, Dubbo, 2830, New South Wales, Australia, csu.edu.au

## Abstract

**Background:**

Research on organisational culture in aged care settings is formative. This study gives primacy to the voices of registered nurses (RNs) who are integral to clinical and managerial leadership in rural and regional aged care contexts in Australia.

**Objective:**

To describe and analyse the reflections and understandings of RNs who are or have been employed in residential and community aged care settings in rural and regional Australia on optimising organisational culture and cultures of care.

**Methods:**

This manuscript reports on a component of a larger study with 14 participants. Five RNs at the board, executive and clinical nurse consultant level employed in residential or community aged care settings in rural or regional Australia during 2017–2024 participated in semistructured interviews. The interview questions were guided by appreciative inquiry, and this facilitated a strength‐based approach to discussion on organisational culture. Data were analysed using critical discourse analysis informed by Michel Foucault, which is concerned with an examination of the nature and utilisation of power as a relational force in social systems.

**Findings:**

The participants, reflecting on their experience in rural and regional contexts, viewed strong nursing leadership as integral to person‐centred cultures in aged care settings. Nursing leadership incorporates nurses working to full scope of practice in settings that support postgraduate study and research and innovation. Interwoven through these perspectives is evidence of Foucault’s concepts of governmentality and disciplinary structures informing nursing practice. Power is both a repressive and productive force that shapes professional relationships and the capacity for RNs to be agents of change. A clear separation of the operational and governance arms of aged care organisations was viewed by participants as necessary for effective management, while highlighting the need for more RNs to contribute in roles at the board and executive level.

**Conclusions:**

This study underscores the opportunity for professional nursing bodies, government agencies and the aged care sector to work together to require RN representation on boards of aged care providers; to develop policies that enable RNs to work to their full scope; and to strengthen mentoring, career pathways, and financial assistance for nurses pursuing further education. RNs working in aged care also emphasised the importance of building a research‐focused culture that drives improvement and innovation, noting that staff education is essential for enabling meaningful involvement in research and project work.

## 1. Introduction

High‐quality care for older people is intrinsically connected to organisational cultures characterised by person‐centredness, where older people and the well‐being of staff are equally valued. Registered nurses (RNs) are central to change processes in aged care settings; however, research exploring the understanding of RNs in relation to organisational culture, particularly in regional and rural contexts is emergent [1].

A rapidly ageing population has implications for the delivery of residential and community‐based services to older people. The number of people over the age of 60 years is expected to double to 2.1 billion people, and it is estimated that the number of people over the age of 80 years will triple to 436 million people by 2050 [2]. The World Health Organization [3] has also identified a limited capacity for the delivery of person‐centred integrated care to older people and access to long‐term care in residential and community environments. A significant cause of limited capacity in the aged care sector is a shortage of care workers and nurses [4] in the Organisation for Economic Cooperation and Development (OECD) countries [5].

In Australia, it is projected that by 2025 there will be an undersupply of 17,551 full‐time equivalent RNs in the aged care sector and a geographical maldistribution impacting the delivery of services in rural and remote areas. These estimates consider legislative requirements regarding mandatory staffing requirements in residential aged care homes [6].

Cameron and Quinn [7], in synthesising multiple perspectives on organisational culture, describe it as the ‘social glue’ consisting of the ‘values, assumptions and expectations’ of members of a group or organisation. In health, workplace cultures are subsets of an organisational culture existing in clinical units or departments, or in groups of health professionals such as nurses or allied health professionals [8]. Cultures of care, another subset of organisational culture, refers to ‘norms of caring behaviour, practices of care and modes of relating’ in a group [9]. Employees are often unaware of some facets of organisational culture, and other aspects are often not discussed, for example, ‘insidious’ racially motivated discriminatory treatment of healthcare professionals [10]. However, organisational culture is none‐the‐less powerful, providing guidelines that promote social cohesion [7, 8]. In this study, organisational culture is examined in the context of residential aged care homes, and community‐based services that provide care to older people living in their own homes. Culture of care pertains to relationships between staff members at different levels of the organisation.

A positive organisational climate, characterised by respect, loyalty and trust, has been found to enhance the retention of nurses employed in acute hospitals [11]. Similarly, another systematic review undertaken by Finn et al. [12] found that a strong safety culture results in increased job satisfaction and reduced turnover in multiple employment groups. Furtado et al. [13] undertook a scoping review and concluded that organisational cultures orientated towards patient safety and quality are more likely to support evidence‐based approaches to care. Research has highlighted that the implementation of new workforce models in aged care is influenced by organisational culture, particularly an openness to change and innovation [14].

In Australia, the regulator of the aged care sector, the Aged Care Quality and Safety Commission (ACQSC) [15], identified organisational culture as a driver of innovation, results and ‘consumer‐centred care’. Råholm and Heggdal [16] linked positive organisational cultures in aged care to a higher level of resident and staff health and well‐being. An integrative review by Magee et al. [1] reported on 17 studies, and only two specifically examined organisational culture in rural aged care settings. The broader themes distilled from all 17 studies was that nurses viewed organisational cultures in aged care settings as distinctive from other health environments. The leadership of nurses was viewed as integral to creating person‐centred cultures in a market‐driven sector [1]. This reflects a systematic literature review identifying that research into organisational culture has occurred primarily in preventative and curative healthcare contexts rather than in environments such as aged care [17]. An integrative review by Churruca et al. [18] concluded further research into the conceptual understanding of organisational culture and how best to facilitate culture change in aged care settings is required. This study responds to the dearth of research by using an innovative approach informed by appreciative inquiry and Foucauldian‐style critical discourse analysis (CDA) to explore organisational culture from the perspective of RNs in rural and regional aged care settings in Australia.

The aim of the study was to describe and analyse the reflections and understandings of RNs on optimising organisational culture and cultures of care who are or have been employed in residential and community aged care settings in rural and regional Australia.

The research questions (RQs) are as follows:

• 1a. What are the features of workplace cultures in aged care?
• 1b. What features are specific to rural and regional areas?
• 2. What organisational structures and processes influence workplace cultures?
• 3. Do organisational mission and values impact workplace cultures? If so, how? If not, why not?
• 4. How is the concept of ‘care’ demonstrated in workplaces, specifically between staff members?

## 2. Research Design

### 2.1. Study Design and Setting

The findings reported in this manuscript are part of a larger study that also included RNs in direct care and leadership positions in facilities. This paper focuses on RNs in board, executive and clinical nurse consultant positions. This study was approved by the Charles Sturt University Human Research Ethics Committee, approval number H23884.

### 2.2. Sampling and Recruitment

**FIGURE 1:**
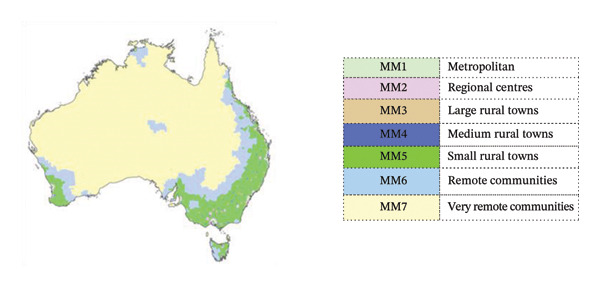
Map of Australia defined by Modified Monash Model (MMM) areas. MMM 2023, published in March 2025 by the Department of Health, Disability and Ageing, available from Digital Atlas of Australia https://digital.atlas.gov.au/datasets/digitalatlas::modified-monash-model/about, © Commonwealth of Australia, used under creative commons attribution 4.0 https://creativecommons.org/licenses/by/4.0/.

Participants in this study were employed in Modified Monash Model (MMM) areas MMM2–MMM5 (regional centres, large rural towns, medium rural towns and small rural towns) in the past 7 years, from 2017 to 2024. The MMM is used by the Australian Government Department of Health, Disability and Ageing to demarcate locations as metropolitan, rural, remote or very remote, and to inform workforce planning [19]. Figure 1 shows a map of Australia coloured to show MMM areas.

This study used a purposive, homogenous sampling method in order to recruit participants with the desired characteristics [20]. Social media and professional networks, such as the Australian College of Nursing and Australian Association of Gerontology, were used extensively to advertise the study. The advertising material and participant information sheet included the names of the research team and contextualised the project as a PhD study.

Participant information and the consent form were accessed and completed electronically. One person completed the consent form but was not interviewed due to family illness. The PhD Candidate (the ‘Candidate’), who was not previously known to the participants, contacted each participant to arrange an interview.

### 2.3. Research methods

This study was informed by two approaches. Appreciative inquiry underpinned the interview questions (IQs). The analysis of the interview transcripts was grounded in CDA drawing on the work of Michel Foucault [21, 22], which connects the discursive statements of actors to the power relations of the site or workplace in which they act or are constrained to act.

The open‐ended IQs facilitated reflection and discussion on the participants’ experiences in nursing prior to and, while working in aged care settings, their understanding of aspects of organisational culture and the conditions to create and sustain positive change [23, 24]. Appreciative inquiry reframes a deficits‐orientated approach into positive assumptions about people, workplaces and organisations. By identifying the strengths of a workplace or organisation, these strengths or ‘generative potential’ can be leveraged and a change process enacted [25, 26].

Similar to appreciative inquiry, the ontological assumption of CDA is that reality is socially constructed and dynamic. Language is pivotal to how reality is created – ‘the way we speak about things constitutes the way we see them’ [27]. For the French post‐structuralist philosopher Michel Foucault, discourse was a way of understanding the social organisation of society through observing the dominant institutions and systems and how they engage with and utilise power [28]. Foucault viewed power as omnipresent, ‘always already there’ and relational in nature, ‘acting upon individuals as they, in turn, act upon others’ [29, 30]. While power can be used to dominate and oppress, it can also be productive, a catalyst for action, transformation and resistance [31, 32].

Four types of power delineated by Foucault are considered in the data analysis. Disciplinary power seeks to regulate individuals and groups through surveillance, control of time and activities and the classification of behaviours as acceptable or ‘abnormal’ [33, 34]. Pastoral power, sometimes defined as a type of disciplinary power, has as its primary concern the physical and psychological well‐being of its subject, overseen by a beneficent figure guiding people towards an endpoint determined as being for their own good [29, 35]. Biopower governs populations by shaping conditions that influence collective behaviour without requiring total individual compliance [36]. Finally, technologies of the self encompasses practices individuals undertake to transform themselves, often in pursuit of ethical, spiritual or professional ideals [37]. These technologies can also serve as mechanisms of resistance, enabling individuals to challenge dominant power structures and form an ‘unsubjugated’ self [37]. For Foucault, the productive and resistant elements of power are neither straight‐forwardly ‘good’ nor ‘bad’ but dependent on context. In this case, we analyse how cultures of care are interpreted by participants as enabling or constraining their actions.

### 2.4. Data Collection

The IQs were designed to elicit information aligned with the study’s aim and RQs and allow space for the dialogue between the Candidate and participants to develop in a natural manner, and for the Candidate to respond reflexively to the evolving discussion [38]. The IQs were pilot tested in a recorded interview with a colleague with substantial professional experience in the aged care sector. Feedback was provided on the Candidate’s approach and the IQs. The feedback was discussed with the supervision team and refinements were made.

Each interview commenced with an informal discussion where the Candidate briefly discussed her professional background and interest in the research topic. Interviews were conducted between January and August 2024 via Microsoft Teams, which provided a secure platform and enabled generation of the interview recording and transcript. Author 1 conducted all interviews. The average interview time was 103 min (1 h and 43 min). There were no repeat interviews. Each participant was asked the following questions, after which the conversation continued in an unstructured manner if the participant wished:

1. Can you tell me about your experience in nursing prior to entering aged care?
2. Thinking about your experiences, could you tell me about the aged care environments in which you have worked?
3. Can you describe how the mission and values of your current/past organisations influence/d how people work?
4. In an ideal world, what would you envisage as the ultimate workplace culture of care?
5. What structures and processes would need to be in place for your vision to become a reality?
6. How would you sustain this positive approach to improvement within the organisation?

**FIGURE 2:**
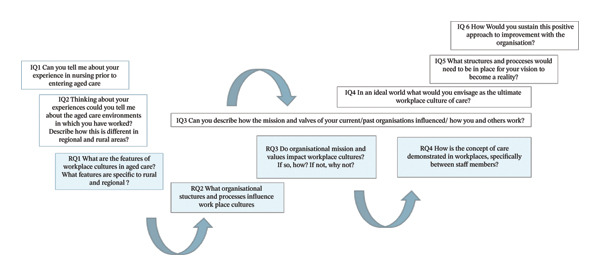
Links between the research questions, labelled RQ, and the interview questions, labelled IQ.

Figure 2 shows the links between the RQs labelled RQ and highlighted in blue, and the IQs, labelled IQ. Note that IQs three and four are linked to more than one RQ. In this study, the notion of care is explored in the context of cultures of care which centres on relationships between staff members working in aged care contexts.

This manuscript focuses on findings from 5 interviews from a larger study with 14 participants. The criteria used to assess data saturation were firstly when interviews were conducted with RNs who sufficiently exemplified a breadth and depth of experience in the aged care sector. Secondly, that the data obtained was of sufficient quality for an analysis to be undertaken and that the conclusions made would be well grounded and robust [39].

### 2.5. Data Analysis

Transcripts were imported into NVivo 12 [40] following a process of familiarisation that included reading and discussing the transcripts as a team, which contributed to the inter‐reliability of the analysis process. The transcripts were organised into nodes according to topic and interview participants.

The second stage of the process was a three‐part Foucauldian‐informed CDA guided by Powers [41]. This entailed studying the video recording of each interview and transcript in‐depth and creating a commentary utilising a genealogical, structural and power analysis. These individual commentaries were shared with and discussed at length with the supervision team. Powers [42] advised that Foucauldian style discourse analysis interprets ‘patterns, rules, assumptions, contraindications, silences, implications and inconsistencies’ in addition to identifying how people function in their social world and how this impacts power distribution.

In the context of this study, the first component of the discourse analysis was the genealogical analysis. This analysis had a historical focus, examining participants’ earlier nursing experiences and how these shaped their current understandings of organisational culture and power [41]. Secondly, the structural analysis included two axes: authority and justification. The axis of authority examined who is permitted to speak within the discourse, and who holds legitimacy and authority and what language is sanctioned. The axis of justification focused on technologies of power, specifically disciplinary power and biopower, requiring close examination of each transcript to understand how participants experienced these forces within aged care settings [41, 43].

The power analysis is considered together with the structural analysis and examines relationships between individuals or groups that enforce or challenge dominant discourses. Technologies of the self as a mode of resistance were also addressed in the power analysis [41].

## 3. Results

### 3.1. Overview of the Participants

This paper focuses on the perspectives of five RNs at the board, executive and clinical nurse consultant level employed in residential or community aged care settings in rural or regional Australia (MMM2–MMM5) in the past 7 years, from 2017 to 2024. Some participants also drew on relevant experience in metropolitan and remote contexts. To protect participant confidentiality a pseudonym was used, and some details of participants’ professional profiles have been altered. The name and professional background of the participants are given in Table 1.

**TABLE 1:** Name (pseudonym) and professional background of the participants.

Name	Professional background	Professional profile
Kay	Board member	Kay has a strong history as a health service executive, and more recently as a board member of a faith‐based not‐for‐profit aged care facility.
Jilly	Manager, non‐government organisation	Jilly has spent her career working in regional, rural and remote areas. These experiences inform her role in a non‐government organisation, which focuses on improving the experience of staff and residents in community and residential aged care across a variety of demographics.
Ana	Executive manager, clinical services, residential and community‐based aged care service	Following a career in leadership and management in the acute care sector, Ana moved into aged care in an executive nursing role in a large rural facility where aged care was co‐located with other services.
Cate	Clinical nurse consultant (aged care) in a community‐focused role	Cate worked extensively in the areas of gerontology and psycho‐gerontology. Later, Cate worked in as a clinical nurse consultant in gerontology in community‐based roles in remote Australia.
Patricia	Clinical nurse consultant (palliative care) for a residential aged care provider	Patricia covers all aged care facilities in the state, working in a hybrid face‐to‐face and online model. Her work centres on assessment, planning and evaluation of end‐of‐life care and supporting nurses in direct care roles through education.

### 3.2. Findings From the Discourse Analysis

The three stages of the analysis illuminate the data within the framework of the RQs and focus on the intersection of organisational culture, cultures of care and power relations in aged care settings. The genealogical analysis is presented in one section. The structural and power analyses are presented for each participant.

#### 3.2.1. Genealogical Analysis: Formative Career Experiences

Reflection on earlier experiences in nursing highlighted workplaces, teams and roles that shaped the participants. Individual participants provided differing levels of detail about previous experiences and the influence of these on nursing practice in later stages of their careers. Jilly’s professional identity after 15 years of practice was a ‘rural and remote slash regional nurse, then midwife’. Patricia trained in the hospital system and then discovered a love of learning in postgraduate study, which has extended across the arc of her career. Kay trained in a large metropolitan teaching hospital and spent time working in acute care settings. Kay returned to work after having children and commenced part‐time employment in an aged care facility in a rural town. Kay described the lack of access to basic equipment such as urinals and washbowls as ‘very tricky’ and acknowledged that this impacted the standard of care for residents, although this situation later improved.

Jilly provided an outline of her experience as a director of nursing in a remote facility. Like Kay, Jilly’s use of language alluded to significant issues, but little detail was provided:

*‘And I suppose just, umm it at the time it was a very political place to work and it really didn’t align with I had four small children* … *so the work that I was having to do and the pressures that were on the role just didn’t align with my personal responsibilities …’*

Cate also trained in the hospital system and commenced her career in an acute care setting. A point of difference from the other participants was Cate discussing this era of her professional life in detail, describing the culture of the multidisciplinary environments that characterised her earlier experiences:

*‘So it was collaboration. It was trusting each other’s abilities, judgements and knowledge and skills. Umm. Working together and understanding them…the reward is, the reward is also about getting those people safe. And no harm coming to them’.*

Throughout the interview, Cate compared her earlier experiences to more contemporary roles and described the challenges of working in teams that she felt were less cohesive.

#### 3.2.2. Structural and Power Analyses: Clinical Expertise and Leadership

Unpinning the structural and power analyses was the participants’ expertise as clinical and managerial leaders, which imbued the participants with the authority to speak on their experiences. Participation in this study can also be viewed as an act of resistance to forces that impede or obstruct the participants’ capacity to function effectively in their professional roles, for example, the boards of aged care providers. Also evident was resistance to larger entities perceived to impact not just individuals but all nurses working in aged care, such as the regulator of the aged care sector, the ACQSC [41, 43].

##### 3.2.2.1. Kay

In discussing the ACQSC, Kay used the descriptor ‘the bureaucracy’, describing them as ‘the very people that are laying down the law’ and their inefficiencies as ‘staggering’ and ‘incomprehensible’. These words directly challenge the credibility of a large government organisation and reflect Kay’s own professional authority and power.

Kay’s experience as a board member offered a valuable and unique insight into how power operates to constrain individuals and organisations. She described the ACQSC as an omnipresent and omnipotent force influencing every person and activity within the facility. The ACQSC functions, ‘resolving, accrediting, monitoring, administering, revoking and regulating’ along with its compliance and enforcement actions (Aged Care Quality and Safety Commission, n.d), reflect a surveillance‐based model of governance. This aligns with Foucault’s concept of governmentality and panopticism, where institutional oversight is maintained through technologies, such as websites, electronic forms and submission portals [44–46].

A defining feature of the interview was the influence of Kay’s religious faith on her role as a board member. This was demonstrated through Kay’s determination to make ethically sound decisions and the sense of commitment and responsibility to all who lived and worked at the facility. This strong sense of responsibility for the well‐being of staff and residents is arguably a form of pastoral power, which is informed by the Christian story of the Good Shepherd, who cares for his sheep at the expense of his safety [29, 35].

Kay’s admiration and respect for those who work in aged care was evident throughout our conversation, and illustrated by the following quote:

*‘The one thing I’d like to say is just to recognise and commend the people that work in aged care and every day, you know, from the managers all the way down, you know, all the all the jobs within aged care that keep places ticking over. I think they do an incredible job’.*

##### 3.2.2.2. Ana

Although Ana declared she was not interested in ‘politics’, her professional self is arguably moulded by the relational aspects of power. Foucault ‘observed that the construction of self (subjectivity) … is created by the influence of multiple forms of power’ [29], p. 559.

Ana identified misuse of power by non‐nursing colleagues and attempts to undermine her decisions several times during the interview. Her anger at this was palpable but she remained circumspect in her use of language. Her response to this misappropriation of power was RNs assuming leadership in the aged care sector.

Ana found the board members to be arrogant and condescending, particularly those who were paid consultants. Ana had to be very careful as to how she managed these relationships because of their professional power. Ana stated that there had been four executive nurses prior to her, and the high turnover was partly due to interference ‘not coming from the CEOs (chief executive officers) but coming from governance’. Ana further noted that

*‘it’s not transparent, and those people can make you or break you. None of them (the executive nurses) lasted more than three months and that’s because they couldn’t bear Big Brother watching everything you do’.*

Ana viewed board and executive staff as obstructive to the advancement of RNs and particularly RNs at the executive level. Ana’s exploration of the methods to increase leadership of RNs can be interpreted as active resistance to the dominant power structures in aged care. It also challenges a discourse that nursing as a profession is devoid of power to challenge the dominance of other professional groups [29, 47].

##### 3.2.2.3. Jilly

Jilly spoke with great passion about creating organisational cultures where RNs can thrive, working to their full scope of practice and leading teams. Like Kay, Jilly’s role included the capacity to exercise pastoral power, guiding the ‘flock’ on a journey, in this case a process of organisational change [35]. Jilly described talking to the staff, ‘understanding what they feel at the moment … where are you at the moment’? She then described a process of education and skills development centred on organisational leaders, followed by working with teams in workplaces. The intent of utilising this power was beneficent, ‘it’s about educating and then following up and really holding them to account’. The change process led by Jilly was for the ultimate good of the staff and residents of the facilities. This discourse aligns with pastoral power being utilised for the good of the collective, but the welfare of the individual is still important. Jilly noted the importance of giving individual RNs ‘permission to be their authentic selves’ and to ‘speak up’ in a ‘safe space’ [48].

In addition to employing the language suggestive of pastoral power, Jilly also spoke the language of corporate environments and neoliberalism, which she used to describe the priorities of the for‐profit aged care providers. The ‘wants and needs’ of the customer mentioned by Jilly are preeminent in the context of for‐profit aged care providers [49]. The word ‘customer’ when used in this way is, according to Holborow [49], ‘endowed with a semi‐reverential status’. Jilly also used coded language, for example, ‘reporting structures or the hierarchy or staffing is very lean in for‐profits’. Jilly is suggesting that for‐profit providers may have fewer staff as this will decrease costs and increase profits.

The process of organisational change encompasses the transformation of individuals. Jilly spoke about RNs becoming their ‘authentic selves’ and developing skills to lead their teams in an environment of psychological safety. Mitcheson [37]; p. 63 emphasises that technologies of the self are situated within a ‘nexus of power relations’. Within the existing power order of the organisation, Jilly, as a kind of double‐agent, enacts the will of the ACQSC and the organisation, and also creates the conditions where resistance to organisational power is possible, through RNs becoming more authentic as people through activities such as reflection, discussion and skill development, and ultimately taking their position as leaders [37].

Jilly noted a change in how the accreditation process was undertaken following the Royal Commission into Aged Care Quality and Safety ’the Royal Commission’. She described the accreditation process as ‘very disciplinary’, and the accreditation surveyors had a ‘matriarchal type attitude’ and gave Jilly the feeling that ‘they were out to find something, anything’. The change in the accreditation surveyors to a more punitive stance following the Royal Commission is reflective of their position as ‘instruments of governmentality’ [50]. Jilly supports RNs working to full scope of practice in aged care even as she acquiesces to the role of the state’s oversight of the aged care sector.

##### 3.2.2.4. Cate

During the interview, Cate reflected on a long career where she built expertise in specialist gerontology roles in inpatient and community settings. She spoke of an experience in her career that caused her great distress, even though it occurred several years ago. Cate worked as an RN in a residential aged care facility during the transition from full time work to retirement. During this time, she experienced issues related to how RNs scope of practice is perceived in an aged care context.

Health professionals engaging in (self‐) surveillance and reporting of colleagues for deviations from policy are indicative of the scope of practice as an aspect of disciplinary power. Cate believed that she was self‐regulating as a professional and following what she understood to be best practice [51]. A meeting with the nurse unit manager (NUM) resulted in what she interpreted as her scope of practice and her autonomy as an RN (a contested term in the milieu of clinical governance) being curtailed [51]. Thus, Cate felt an acute loss of professional power. The professional codes and guidelines that delineate among other things the scope of practice of RNs are a discourse that provides a necessary framework for nursing knowledge but also arguably serve, along with organisational policy and procedure, to constrain the practice and power of RNs in aged care settings [52–54]. The disclosure of this experience in the interview can be viewed as an act of resistance to disciplinary power by Cate, aiming to reclaim her professional power and her identity as an RN [55].

##### 3.2.2.5. Patricia

Working as a consultant across her organisation, Patricia has developed an insight into, for example, how geographical location and the staff profile impacts how a facility operates. Patricia alluded to some occasional problems when she visits facilities, ‘things won’t be as they should, or in good order’; however, she is careful in her choice of language and the level of detail in which she describes these issues. The ability to be circumspect is also characteristic of Kay, Ana and Jilly. While this is a component of their personalities, it is also indicative of their professionalism and the high value placed on confidentiality by the profession [56].

Patricia does not regularly interact with her organisation’s board but values their engagement with clinical governance reports. With a focus on ‘each aged care consumer’, clinical governance aims to ensure safe, high‐quality care and positive outcomes. This is achieved through an ‘integrated set of leadership behaviours, policies, procedures, responsibilities, relationships, planning, monitoring and improvement mechanisms’ [57]. Clinical governance exemplifies Foucault’s concept of a disciplinary society, where monitoring and reporting are used to achieve specific outcomes [51]. In modern capitalist contexts, this is reflected in the prevalence of audit practices, tools for ‘governing at a distance’ and evaluating expert activities such as those of health professionals [51], p. 163.

## 4. Addressing the Research Questions

### 4.1. Research Question 1a: What Are the Features of Workplace Cultures in Aged Care?

The features of workplace cultures in aged care settings were referenced in the participants’ exploration of previous and current professional roles. Kay emphasised the importance of the mission and values across the breadth of work undertaken in a residential aged care home. In Kay’s experience as a board member, value statements were more influential than mission statements, as many care staff were from non‐Christian backgrounds. Kay spoke of her admiration for the second CEO she worked with, a man of faith from a non‐Christian background. The organisational mission and values were apparent in how the CEO approached his role – ‘he just totally got it and totally embraced it and totally supported it and communicated it to all the staff’. She identified that his leadership improved staff retention, as staff felt respected and understood. Personally, Kay appreciated the honesty, transparency and integrity that he bought to the role.

The appreciation that the board members such as Kay and the CEO had for the staff was expressed through a celebratory barbecue, for example, when accreditation was successful and the annual staff award night. The managers were taken to dinner by the board at Christmas and presented with their Christmas bonuses. The board attended these events as a way of showing their appreciation and support to the staff which contributed towards the development of a positive workplace culture. However, Kay emphasised that that this did not replace the need to establish and maintain relationships with staff through being a visible presence in the facility, which she visited frequently to talk to both staff and residents.

In Jilly’s experience, person‐centred care was a core philosophy of both for‐profit and not‐for‐profit providers. Respect, compassion, teamwork and a commitment to learning were organisational values noted by Jilly as being consistent across her employers. These values, when enacted across the entire organisation, resulted in a ‘feeling of support and being cradled for want of a better word. I don’t know out how else to say it. A feeling of family’. This support included board members and the general manager visiting facilities with a genuine interest in hearing about successes and problems and contributing in a meaningful way to finding solutions. In reflecting on a previous role as manager of a flexible aged care centre in a remote context, Cate described the staff as ‘very, very much about themselves’. A key issue for Cate was nursing and care staff not presenting for shifts and leaving the community unexpectedly, suggesting an organisational culture not based on concepts such as respect and accountability – a failure of staff to exercise appropriate governmentality through self‐management. Absenteeism resulted in Cate working a great deal of overtime due to lack of staff. Cate admitted that this role caused her enormous stress, impacting her physical and mental health, ending with her resignation after only a few months – ‘I lasted 6 months … nearly killed me, lost 10 kilos. You were everything, the butcher, the bloody vet, the cook, the cleaner …’

### 4.2. Research Question 1b: What Organisational Structures and Processes Influence Workplace Cultures?

Patricia, Cate, Jilly and Ana also identified a range of issues working in aged care in rural and remote contexts related to staffing, access to services and in some cases equipment. Patricia reported a need for ‘fly‐in, fly‐out’ contracts due to recruitment and retention issues for nursing and allied health staff in some rural aged care facilities. These facilities also on occasion faced challenges in accessing equipment, for example, syringe drivers. Patricia labelled the experience of RNs working with a lack of access to general practitioner (GP) services as a ‘burden of care’.

Jilly discussed the challenges of working in a remote environment as an RN in aged care, highlighting the complexity of the skill set required. Advanced assessment skills, the capacity to work with clients with multiple chronic diseases and for whom English was not their first language, ‘the lack of GP’s … the more remote you go … you have clinics, not hospitals. So, there’s a there’s a pressure on registered nurses to deliver an extended scope of care’. Ana described ‘working with limited staff, having to be all things to all people and manage complex crises and situations with no GP down the road or anything like that is very difficult’. For Cate, the capacity to establish and maintain good relationships with colleagues was imperative in community‐based roles, along with the ability to work autonomously. From Ana’s perspective, there was a lack of understanding from board members who were not familiar with the organisation or the complexities of rural aged care settings:

‘you imagine what it’s like when you are trying to run an organisation and you’ve got somebody who never comes to your organisation, only comes in via video conference and then starts to actually have an active role and chairing the clinical quality meetings’.

In spite of these challenges, participants identified that aged care services in rural and remote areas need to be adaptable and innovative in order to meet the needs of older people. Jilly’s organisation had just established a telehealth service, although some services such as palliative care were still difficult to access. In the future, Jilly anticipated RNs providing vaccinations in aged care settings. Palliative Care Needs Rounds link rural residential aged care facilities to palliative care clinicians. The aim of this service, according to Patricia, was to improve the quality of care at end of life and reduce transfers to acute settings.

### 4.3. Research Question 2: What Organisational Structures and Processes Influence Workplace Cultures?

Kay and Ana shared their experiences with the boards of aged care providers. Kay discussed being a board member and later chair of the board of an aged care facility. Ana, as executive manager of clinical services, interacted with the board and other executive‐level staff frequently.

Kay and Ana emphasised the necessity for strict separation between the strategic focus of the board and the roles that impact the daily life of residents [58]. There was tension between Kay’s role as a board member and her role as an RN. Kay felt challenged at times to remain within the boundaries of her role. ‘I had to constantly pull myself back because it would have been so easy for me just to have acted like a DON (director of nursing) or a CEO’.

Kay and Ana agreed that effective boards of aged care providers should include GPs, allied health professionals and RNs. The presence of academics would also be valuable. The board should have a clinical arm and there should also be, according to Ana, ‘those people that understand economics, that understand how they’re spending the dollar, that understand quality, that understand the values of the organisation and aren’t afraid to speak up’. Ana emphasised that paid external consultants often destabilised the effective management of an organisation. Board roles should have a ‘rigorous selection process’ with appropriate training for appointees so that they understand their role and matters such as conflict of interest which can be a concern in rural communities.

The participants saw great worth in a specialist gerontology workforce in aged care, and the presence of gerontology nurse practitioners across the aged care sector, particularly in light of the lack of GPs servicing aged care facilities in rural and regional areas. Ana was dismayed at gerontology nurses unable to work to the full scope of practice because of organisational policy that didn’t facilitate comprehensive nursing assessment in, for example, acute deterioration. Ana described this as nurses being ‘dumbed down’. Ana also identified that there was a lack of policy to support interventions such as insertion of in‐dwelling urinary catheters by RNs. This highlights the need for research that contributes to the evidence‐base so that urinary catheters are used appropriately in residential and community care, and so that the rights of older people are respected [59].

### 4.4. Research Question 3: Do Organisational Mission and Values Impact Workplace Cultures? If so, How? If Not, Why Not ?

Kay and Patricia both emphasised the necessity for the integration of mission and value statements across the breadth of work undertaken by organisations. In Kay’s experience, values‐based leadership improves retention, as staff feel respected and understood. Patricia recalled that the necessity for employees to embody mission and values in their work is reflected in the employee recruitment, on‐boarding and performance review processes.

Ana observed that boards of not‐for‐profit aged care providers often prioritised budgets and reputational concerns over values. She believed that adherence to values was more about public image than genuine commitment. In terms of how the mission and values influenced the staff in direct care roles, Ana’s perception was that values were adhered to so that the reputation of the facility was maintained in the community, ‘if the community think there is quality care, then the board are happy, and you never embarrass the board’.

Kay reflected on the mission and values of her organisation, which were firmly grounded in Christian ethos, ‘the mission was to care for people based on the life and teaching of Jesus Christ’. Like Kay, Patricia recognised a congruence between the mission and values of organisations she had worked in and nursing’s professional values, ‘the premise is that you’re expressing Christ’s love, so it’s that it fits very much with the nursing philosophy of caring for others’.

Kay noted that the alignment between the organisational values and the values embedded in the nursing professional codes and guidelines supported the work of the RNs in providing person‐centred care. Psychological safety, also described by Jilly as a ‘nurturing’, is an integral component of a positive organisational culture characterised by respect, compassion, teamwork and a commitment to learning. Ana recognised the centrality of person‐centred care to older people’s quality of life and the necessity for this philosophy to underpin models of care [60].

Cate, drawing on her professional values of equity, social justice and respect emphasised the importance of open communication, trust and collaboration. She was critical of superficial value statements, particularly in culturally sensitive contexts, noting that genuine relationships must precede trust and engagement.

‘I don’t care where you work and as you know, I can talk to you until the cows come home and this is the constant thing … working with Aboriginal people is a fine example of this. If they think you’re bullshitting they just go &∗%^ off and just turn off. If you don’t have a relationship first, then they’ll just say yes, yes, yes, yes, and go away. And it’s the same thing with any any, any human really’.

### 4.5. Research Question 4: How is the Concept of ‘Care’ Demonstrated in Workplaces, Specifically Between Staff Members?

This question was explored in the interviews through the concept of cultures of care. In order to create the optimum culture of care, participants highlighted committed and visible leaders modelling collaboration and the creation of positive relationships. In creating an optimum culture of care, Kay drew on her own philosophy as a nursing leader, asserting that her first priority was always to care for her immediate team, and that this would hopefully cascade down throughout the larger team. Kay applied this same philosophy as a board member,

*‘If it genuinely is a great environment right from the top and, you know the board is cohesive and our board certainly was cohesive, there was no, there was no conflict at all within the board and you’ve got a really great relationship with your chief executive. And then there’s no reason that that positive approach can’t filter down through all the components within that organisational structure …’*

Maintaining an optimal culture of care, for Kay, was contingent on relationships between staff at various levels of the organisation. Kay highlighted that the board knew the managers on a personal basis. In her case, Kay had met most of the staff, and that overall, board members were recognisable to staff in the facility. Kay identified the challenges of creating cohesion and loyalty due to a highly mobile workforce and underscored the importance of drive and determination to create a positive environment. In the case of the CEO, Kay felt that he had succeeded in this as evidenced by improved staff retention and that this was noted by the residents. This was evidenced by a conversation with a resident who said to her that ‘the staff that look after us every day of the week are the most important to us, and if they are looked after then we’re going to be happy’.

When asked about the features of an optimal culture of care, Ana and Patricia underscored that the facility leadership needs to be visible and accessible to staff and ‘lead on the ground’, demonstrating commitment to good clinical outcomes and to positive relationships with staff and residents. Jilly described a practice development approach to creating optimum cultures of care across the facilities and in her organisation. The philosophy of person‐centred care provided the framework for the intended culture of care. This person‐centredness ‘was for everybody, our staff as well as our residents’.

Jilly observed that not‐for‐profit providers provided more educational support, while for‐profit organisations often had lean staffing structures and limited managerial or clinical governance support. Jilly described this as a ‘crush’, where RNs were overwhelmed and felt undervalued, often ‘stuck behind a trolley’ administering medications. This pressure was exacerbated by service managers from non‐clinical backgrounds, such as hospitality or human resources, who lacked understanding of the nursing scope of practice. Conversations with boards and executives in for‐profit settings had a distinctly ‘corporate’ tone, focusing on budgets, Australian National Aged Care Classification (ANACC), key performance indicators and complaint management.

Participants agreed that RNs should undertake post‐graduate certificates that include the development of skills in advanced clinical assessment, physiology and complex practice. Ana felt that clinical master of nursing programmes funded by government and developed with significant input from advisory groups from industry were ideal, rather than a theoretical master’s degree. In addition, Cate also identified experiential learning guided by a clinical expert in a mentoring role as pivotal.

Patricia emphasised the importance of mentoring, career progression and financial support for RNs to undertake further study. This will attract graduate nurses into the sector and ‘lift our industry beyond what was the Royal Commission’, which Patricia felt clearly diminished the perception of the value that nurses bring to aged care settings and the lives of older people. The concept of ‘uplifting’ nurses working in aged care was a concept that was important to Patricia and integral to her idea of leadership [59].

Patricia highlighted that a research culture is important as it provides a way of validating best practice. However, she also acknowledged that RNs in aged care need to embrace opportunities to extend their learning and further their career. The change in the health needs of residents in aged care facilities also requires organisations to be responsive and open to innovation. Research was noted by Jilly, Ana and Patricia as being part of a healthy organisational culture. Jilly and Ana felt this was not a priority for providers, particularly those with non‐nursing leaders and boards with a majority of non‐clinicians. Ana noted that research priorities should extend beyond a focus on neurocognitive disorders, as older people have complex and multifaceted needs.

## 5. Discussion

The current status of RNs as ‘disciplined bodies’ subject to a ‘crush’ as participant Jilly described, of various forms of power, is accompanied by a parallel discourse of resistance that seeks optimal organisational cultures underpinned by nursing leadership and person‐centred models of care [61]. There are opportunities for collaboration between professional nursing organisations, government bodies and the aged care sector to expand opportunities for RNs to participate at the board and executive level, and for all nurses to practice to full professional scope, thereby promoting person‐centred environments in residential and community aged care settings. Research is crucial for driving innovation and improving care quality for older adults. Participants stressed the need to remove barriers to research and to introduce incentives such as linking providers’ research involvement to quality indicators.

### 5.1. The Need for Strong Nursing Leadership

The participants identified that the presence of nursing leadership was integral to a positive organisational culture. RNs should be present on boards and at the executive level of aged care providers to provide relevant expertise and along with other board members, model organisational values in such as integrity and honesty [60, 62]. RNs can contribute insights drawn from their experience in aged care to discussions at the board level on organisational culture, for example, an understanding of the influence of workforce subcultures, cultures of care and informal leadership [63].

In Australia, the ACQSC [64] mandated that the boards of aged care providers include at least one member with clinical care experience and relevant qualifications. However, there is a strong case for greater specificity about the breadth and depth of clinical expertise required, especially given the increasing number of residents over 85 and the rising prevalence of frailty and complex conditions [65, 66]. RNs with substantial experience in clinical governance are well‐positioned to contribute meaningfully at board and committee levels.

### 5.2. Person‐Centred Practice is an Antecedent to High‐Quality Care

Ana, Kay and Jilly recognised person‐centred practice as intrinsic to high‐quality care in aged care settings. Person‐centred care can be understood as a philosophical response to the disciplinary nature of biomedical approaches, which fragment the body into systems for examination and treatment, often rendering the patient a ‘docile body’ (May, 1992) [68]. This reductionist view aligns with the Foucauldian concept of the clinical gaze, where medical power is exercised through observation, categorisation and intervention [69, 70], which is also ‘embodied in nursing work’ (May, 1992). In contrast, person‐centred care resists this paradigm by prioritising the individual’s lived experience, relationships and autonomy, reflecting nursing’s commitment to holistic and respectful care [71, 72].

Person‐centred relationships that extend to relationships between staff are a catalyst for human flourishing [73]. Person‐centred relationships are characterised by trust, respect and collaboration; terms used by the participants to describe the features of a healthy organisational culture. [68]. In Australia, the strengthened aged care quality standards, while embracing person‐centredness in the context of older people, do not extend the concept to person‐centred models of care that encompass the flourishing of staff and a ‘healthful’ organisational culture [60, 74]. In a study of the person‐centred characteristics of residential aged care homes in Australia, Seah et al. [75] observed that a primary indicator of a person‐centred environment was mutual regard, respect and engagement including relationships between staff members, and a commitment by the organisation to staff well‐being. However, the current funding models and regulatory frameworks within the aged care sector may impede the provision of person‐centred approaches due to a focus on compliance and funding security. However, greater attention should be given to equipping and supporting the workforce [76].

### 5.3. Working to Full Scope of Practice

The flourishing of staff in person‐centred environments described by Dewing and McCormack [73] suggests nurses in aged care settings working to the full scope of practice in all roles, providing leadership at the facility and organisational levels. This was viewed by participants as a component of a healthy organisational culture and integral to the quality of care received by older people. [53, 77]. Scope of practice refers to the ‘specific activities and responsibilities that nurses are educated, trained, and legally authorised to perform. These activities are shaped by a nurse’s qualifications, registration type, education, relevant regulations, competence, and confidence, ensuring they align with their skills and expertise’ [52].

In Australia, the designated RN prescriber role and the nurse practitioner workforce plan extend the capacity of RNs to provide high‐quality care to older people in aged care settings [78, 79]. This may benefit rural areas where there is a lack of access to GP services. Mentoring and financial support for post‐graduate education is an important part of the framework that supports the expansion of these programs.

Malatzky et al. [80] situated the narrative surrounding the rural GP shortage as part of a dominant discourse in Australia that primary healthcare, which includes aged care, can only be provided by GPs. This silences discussion on the role that nurses and other allied health professionals play in the provision of primary healthcare internationally and reinforces a perception that training more GPs is the only solution to workforce shortages [80]. The support for the nurse practitioner workforce plan [78] by the Nursing and Midwifery Board of Australia is an example of a point of resistance that has changed processes and systems that depend on disciplinary mechanisms.

### 5.4. Research Within the Aged Care Sector

Participants emphasised the importance of research to drive innovation and improve the quality of care for older people. It is essential that barriers to research are removed and that incentivisation initiatives suitable to a quasi‐market sector are implemented, for example, linking provider research participation and outcomes to quality indicators [66, 81, 82]. Organisational culture, specifically the alignment between the strategic priorities of the organisation, the support of leadership and attitude to staff autonomy, all impact innovation and implementation research approaches in aged care. RNs and care staff understand the importance of research conducted in aged care settings. However, further education on the research process generally and the importance of specific projects would benefit the diverse occupational and cultural groups working in aged care and facilitate staff ‘buy‐in’ [83].

Practice development fosters a local approach to workplace cultures as distinct from organisational culture. In aged care settings, the concept of the ‘embedded researcher’ supports quality improvement initiatives, the translation of evidence to practice and collaborative research in partnership with older people and external stakeholders [84]. Practice development’s embracing of learning, development and improvement is congruent with the Foucauldian concept of technologies of the self [85]. Through practice development, RNs can reject their status as ‘docile bodies’ subject to disciplinary power, work to full scope of practice, embrace roles as embedded researchers and assume leadership positions in aged care settings [86].

### 5.5. Strengths and Limitations

A strength of this study is the diversity of the participants which, in conjunction with the breadth and depth of professional experience, added to the richness of the data collected. The utilisation of appreciative inquiry and CDA is an innovative way of explicating both the current challenges of working in aged care environments and the participants’ perspectives on creating healthy organisational cultures.

While the nursing workforce in Australia is predominantly female, a potential limitation is that no participants discussed in this paper identified as male [87]. Another possible limitation of this study is that all the participants were born in Australia and thus the views of RNs educated overseas are not captured.

## 6. Conclusions

Nurse leaders from rural and regional aged care settings agreed that person‐centred organisational cultures reinforced by strong nursing leadership are intrinsic to high‐quality care. They called for coordinated action across professional nursing organisations, government and providers to embed RN representation on aged care boards; implement policies that remove the scope of practice barriers; and expand mentoring, career pathways, and financial support for post‐graduate study. Leaders also stressed the importance of cultivating a research‐active culture that drives improvement and innovation, noting that staff education is crucial for enabling meaningful participation in projects and studies. While the participants recognised the constraining forces impacting their practice, their past experiences and future plans are also a narrative of resistance and the dismantling of barriers so that RNs work to their full potential.

## Funding

No funding was received for this manuscript.

Open access publishing was facilitated by Charles Sturt University, as part of the Wiley–Charles Sturt University agreement via the Council of Australasian University Librarians.

## Conflicts of Interest

The authors declare no conflicts of interest.

## Supporting Information

Additional supporting information can be found online in the Supporting Information section.

## Supporting information


**Supporting Information** A consolidated criteria for reporting qualitative research checklist (COREQ) was submitted with this manuscript as a supporting file to ensure comprehensive reporting of the study [83].

## Data Availability

Research data are not shared.
