# Supplementary material for: Reflections of Nurse Leaders on Optimising Organisational Culture in Aged Care in Rural and Regional Australia: A Critical Discourse Analysis
Source: J Nurs Manag. 2026 May 30;2026:1474638. doi: 10.1155/jonm/1474638 (PMC13240421; doi:10.1155/jonm/1474638)
Supplement: Supplementary file 1 — Supporting Information A consolidated criteria for reporting qualitative research checklist (COREQ) was submitted with this manuscript as a supporting file to ensure comprehensive reporting of the study [83]. [file JONM-2026-1474638-s001.docx]

**This COREQ checklist has been submitted in order to comply with the author requirements of the journal. COREQ is a validated reporting guideline for qualitative studies. Please see reference at conclusion of checklist.**

COREQ Checklist - Reflections of nurse leaders on optimising organisational culture

in aged care in rural and regional Australia: a critical discourse analysis

| **No Item Guide** | **Questions/description** | **Notes** |
| --- | --- | --- |
| **Domain 1: Research team and reflexivity** | | |
| **Personal Characteristics** | | |
| 1. Interviewer/facilitator | Which author/s conducted the interview or focus group? | Deborah Magee |
| 2. Credentials | What were the researcher’s credentials? E.g. PhD, MD | Deborah Magee RN M.Phil  Marguerite Bramble RN PhD  Holly Randell-Moon PhD  Karen Francis RN PhD |
| 3. Occupation | What was their occupation at the time of the study? | The authors are all academics at Charles Sturt University |
| 4. Gender | Was the researcher male or female? | Female |
| 5. Experience and training | What experience or training did the researcher have? | The primary author is a PhD Candidate who has previously completed a Master of Philosophy. She also works as a research assistant. |
| **Relationship with participants** | | |
| 6. Relationship established | Was a relationship established prior to study commencement? | See page 7 |
| 7. Participant knowledge of the interviewer | What did the participants know about the researcher? e.g. personal goals, reasons for doing the research | See page 10 |
| 8. Interviewer characteristics | What characteristics were reported about the interviewer/facilitator? e.g. Bias, assumptions, reasons and interests in the research topic | See page 10 |

| **Domain 2: study design** | | | |
| --- | --- | --- | --- |
| **Theoretical framework** | | | |
| 9. Methodological orientation and theory | What methodological orientation was stated to underpin the study? e.g. grounded theory, | Commences page 7 | |
| **Participant selection** | | | |
| 10. Sampling | How were participants selected? e.g. purposive, convenience, consecutive, snowball | | Page 8 |
| 11. Method of approach | How were participants approached? e.g. face-to-face, telephone, mail, email | | Page 8 |
| 12. Sample size | How many participants were in the study? | | Page 8 |
| 13. Non-participation | How many people refused to participate or dropped out? Reasons? | | Page 8 |

| **Setting** | | | | | |  |
| --- | --- | --- | --- | --- | --- | --- |
| 14. Setting of data collection | | Where was the data collected? e.g. home, clinic, workplace | | Page 10 | | |
| 15. Presence of non-participants | | Was anyone else present besides the participants and researchers? | | | Page 10 |  |
| 16. Description of sample | | What are the important characteristics of the sample? e.g. demographic data, date | | | Commences page 12 |  |
| **Data collection** | | | | | |  |
| 17. Interview guide | | Were questions, prompts, guides provided by the authors? Was it pilot tested? | | | Page 10 |  |
| 18. Repeat interviews | | Were repeat interviews carried out? If yes, how many? | | | Page 10 |  |
| 19. Audio/visual recording | | Did the research use audio or visual recording to collect the data? | | | Page 10 |  |
| 20. Field notes | | Were field notes made during and/or after the interview or focus group? | | | No |  |
| 21. Duration | | What was the duration of the interviews or focus group? | | | Page 10 |  |
| 22. Data saturation | | Was data saturation discussed? | | | Page 11 |  |
| 23. Transcripts returned | | Were transcripts returned to participants for comment and/or correction? | | | No |  |
| **Domain 3: analysis and findings** | | | | | |  |
| **Data analysis** | | | | | |  |
| 24. Number of data coders | | How many data coders coded the data? | | | Discussion commences page 11 |  |
| 25. Description of the coding tree | | Did authors provide a description of the coding tree? | | | N/A |  |
| 26. Derivation of themes | | Were themes identified in advance or derived from the data? | | | N/A |  |
| 27. Software | | What software, if applicable, was used to manage the data? | | | Page 11 |  |
| 28. Participant checking | | Did participants provide feedback on the findings? | | | No |  |
| **Reporting** | | | | | |  |
| 29. Quotations presented | Were participant quotations presented to illustrate the themes / findings? Was each  quotation identified? e.g. participant number | | Discussion commences page 13. | | |  |
| 30. Data and findings consistent | Was there consistency between the data presented and the findings? | | | | Discussion commences page 13. |  |
| 31. Clarity of major themes | Were major themes clearly presented in the findings? | | | | Discussion commences page 13. |  |
| 32. Clarity of minor themes | Is there a description of diverse cases or discussion of minor themes? | | | | N/A |  |

Tong, A., Sainsbury, P., & Craig, J. (2007). Consolidated criteria for reporting qualitative research (COREQ): a 32-item checklist for interviews and focus groups. *International journal for quality in health care*, *19*(6), 349-357. <https://doi.org/10.1093/intqhc/mzm042>
